# Editor's view: What makes a scientist successful?

**DOI:** 10.7189/jogh.16.01002

**Published:** 2026-03-30

**Authors:** Igor Rudan

**Affiliations:** 1 Centre for Global Health, Usher Institute, The University of Edinburgh, UK; 2 Nuffield Department of Primary Care Health Sciences and Green Templeton College, Oxford University, UK

## Abstract

This editorial examines the nature of scientific discovery by studying the characteristics, methods, and tools that have historically defined a ‘successful scientist’. It first considers the traditional hypothesis-driven model of science founded on existing knowledge, testable hypotheses, and experiments based on precise measurements. Through illustrative examples, it distinguishes three types of highly successful scientific contributors: those who formulated novel hypotheses and theories that proved groundbreaking, those who developed transformative measurement tools that enabled experimental testing, and those who managed to do both, even in multiple scientific fields. While success has historically depended on access to knowledge, exceptional creativity, perseverance, and experimental rigor, this editorial reminds that serendipity and timing have also played important roles. It also points to the limits of science when hypotheses remain untestable and theories unfalsifiable. It then reviews modern developments that challenge traditional approaches, such as genome-wide association studies (GWAS), where clear and specific hypotheses are no longer a prerequisite for important novel discoveries, and where discovery can emerge from data mining, rather than from pre-existing knowledge and creative hypothesis. The editorial then progresses onto the emergence of machine-led scientific discovery, using the example of the DeepMind team’s development of AlphaFold – an artificial intelligence (AI) system that accurately predicted protein folding without relying on traditional hypotheses. AlphaFold learned directly from data and revolutionised the entire field of structural biology, bringing a new era of machine-inferred and AI-based science. The described frameworks are then used to analyse the development of an emerging field of science, ‘ideometrics’, based on the theory of the brain’s ‘sense of ideas’, explaining how it could potentially contribute to understanding the purpose of consciousness as a complex trait in evolutionary terms. Being conscious provides an advantage by enabling the brain’s ‘sense of ideas’ to reduce informational entropy of all possible future states of a conscious being to a narrower range of the outcomes that are perceived as more favourable for its survival. Perception of time and space in humans is, therefore, intertwined with their conscious ‘sense of ideas’, both while awake and dreaming, and further research in neuroscience will be required to elucidate these relationships. In conclusion, the editorial offers a comprehensive reflection on how the definition of a ‘successful scientist’ is being substantially reshaped in the 21st century.

In this editorial, I will attempt to explain how scientific work generates and expands human knowledge. Historically, science has advanced through an alternating sequence of major breakthroughs in our understanding of natural phenomena, followed by smaller, refining steps. These numerous incremental steps then contributed to the accumulation of additional knowledge, which was necessary to reach a critical point from which firm foundations for new major breakthroughs could emerge. Progress was often achieved by proposing new hypotheses based on existing knowledge and then testing them after collecting further information to prove their correctness or to reject them as incorrect. This depended on elegantly designed experiments and reliable measurements, which had to be conducted with extreme precision, in accordance with the principles of ethics and scientific integrity. I will consider the key elements of hypothesis-driven science, briefly present the work of the most successful scientists who employed this approach, and reflect on what has historically been viewed as ‘success’ for an individual scientist and how this understanding may change in the 21st century.

Throughout history, many members of the academic community distinguished themselves by drawing upon existing human knowledge and proposing original, insightful, and often counterintuitive hypotheses. These hypotheses helped explain the natural world in a more comprehensive and precise way than previous prevailing beliefs. An original idea, an intriguing question, and the resulting scientific hypothesis have traditionally formed the core of scientific progress and represented only its first step. This step required scientists to acquire deep knowledge of questions that had already been well researched and combine it with personal curiosity and imagination to offer new explanations for natural phenomena. Historically, access to the necessary knowledge was limited to a narrow, privileged segment of society, with men enjoying an incomparably more favourable position than women. However, merely gathering knowledge was not sufficient to become a scientist without also having a creative mind. The reverse is also true: being curious, imaginative, and creative was not enough to become a scientist, as these traits could rarely compensate for a lack of understanding of what was already known. Without a necessary knowledge base, creative individuals could not easily form novel hypotheses that would hold much value among scientists.

Therefore, it seems that, throughout much of human history, only a small fraction of people were in a position to engage in science and contribute to the collective body of human knowledge. If the two prerequisites mentioned above, *i.e.* access to knowledge and exceptional creativity, were both met, then there were additional desirable personality traits for successful scientists. Given that they needed to design and conduct studies to produce the data that might support or refute the hypothesis, this often involved designing elegant experiments and many years of focused and dedicated work. Experiments were frequently hampered by a variety of unforeseen problems, requiring persistence to carry them out as originally planned. They also needed to be conducted ethically, without harming others or causing any harm. After many years of dedicated work, the results would rarely support the proposed new hypothesis. Accepting these negative outcomes required honesty, and some could not resist the temptation to fabricate their results in order to ‘prove’ their hypothesis. Such scientists may have justified their actions to themselves through a firm belief that the enormous effort they have invested simply ‘deserved’ to be rewarded. Most active scientists will understand very well that this is often not the case.

Akin to entrepreneurs, writers, or gold diggers, aspiring scientist need to accept very early that a career in science is highly uncertain and unpredictable, and that it involves a significant element of good fortune, not just personal capability. In an overwhelming majority of experiments ever conducted, scientists invested great efforts only to obtain negative results, which assisted scientific progress in general, but forced them to reject what had initially seemed a highly promising and exciting hypothesis that could advance their careers. Some found this too difficult to accept and, unfortunately, attempted to deceive their peers and the public, falsely claiming to have found solutions to problems [1]. The lesson from such incidents is that any new scientific discovery should be met with a degree of caution in the period immediately after its publication. Only independent replication and verification of experiments, carried out by other research groups, will increase the credibility of any positive result over a prolonged period.

For this reason, true scientific progress can often be assessed mainly in hindsight, after the passage of many years, decades, or even centuries. The most prestigious scientific awards are often granted for breakthroughs and discoveries that have stood the test of time and proven their value to all of humanity. In the next section of this editorial, I will present examples of several prominent scientists who used the traditional approach of hypothesis testing. They were either already recognised for their work, or they certainly deserved to be. The brief selection offered here should help explain why the historical development of science has been driven primarily by hypotheses grounded in existing knowledge and their testing through careful measurement of nature in well-designed experiments.

## SEVERAL HEROES OF HYPOTHESIS-DRIVEN SCIENCE

I will begin this overview with several scientists who proposed hypotheses that directly contradicted the most obvious interpretations of the world around us, formed from the information gathered by our main senses. Their work demonstrated that our senses evolved primarily to serve our daily need to survive, rather than to provide us with a fundamental understanding of the nature that we inhabit. The first two examples are quite straightforward: one obvious interpretation, based on sensory experience, is that the Earth is a flat plane; the other, that the Sun and the Moon both revolve around our flat world at regular intervals. These two beliefs served all people on the planet very well for many thousands of years. We may therefore ask: why did some scientists feel the need to question them, when both beliefs were so obvious and consistent with all the previous human experience? What could humanity possibly gain from declaring them incorrect?

Throughout the history of science, prevailing theories have often been challenged when numerous minor observations began to accumulate and could simply not be reconciled with the widely accepted theories anymore. Such unexpected anomalies often ignited the imagination of a sufficiently critical mass of researchers to set them on a quest for better explanations.

In the case of the ‘flat Earth’, one might reasonably assume that simply observing the Sun or the Moon in the sky would suggest that celestial bodies can be round. However, they could still also be two flat disks, oriented towards the Earth in such a way that they appear perfectly circular from our perspective. The more perceptive among the observers would have noticed that the Moon has phases, and that an increasingly large portion of its surface becomes illuminated by the Sun in a manner consistent with a spherical object. It was, therefore, possible to conclude that the Moon is most likely a spherical object. Still, that did not necessarily imply that the Earth had to be spherical. Those even more insightful might have concluded that, twice a year, the Sun and Moon align in such a way that our world passes directly between them, casting a shadow on the full Moon and causing a lunar eclipse. Naturally, the shadow of our world projected onto the Moon is also circular. Yet this could still be explained if the Earth were a disk, orbited by two other such disks.

Over time, new pieces of information emerged that did not fit with the belief in a flat Earth. One such observation is often attributed to Aristotle (384–322 BC) who, looking far out over the sea, noticed that the sails and masts of approaching ships became visible before the rest of the vessel [2]. This contradicted the explanation of a ‘horizontal’ sea surface and indicated its curvature. Such inconsistencies in theory should lead to the development of alternative hypotheses. Our world might be a sphere, albeit an incredibly large one. In fact, it may be so incomprehensibly vast, from a human perspective, that no one had previously considered that possibility. But how could one design an experiment or gather useful information that could confirm or refute such a radical hypothesis?

In antiquity, the most prominent scholars from Egypt, Greece, and across the Mediterranean sought opportunities to work at the Library of Alexandria. One of them was the ancient Greek astronomer Eratosthenes of Cyrene (276–194 BC). He made an interesting observation during the summer solstice: at noon, vertically built structures, or even long sticks driven into the ground, would cast no shadow at all in the city of Syene in southern Egypt (modern-day Aswan). However, they did cast a small shadow in Alexandria, located further north. Assuming that all rays of sunlight reaching Earth are parallel to one another, this indicated that the surface of the Earth must be curved. Furthermore, simply knowing the distance between Syene and Alexandria would make it possible to calculate the Earth’s entire circumference [3]. Eratosthenes’ estimate is believed to have been astonishingly close to the modern value of 40 041 km. However, even after his experiment, the spherical Earth was not proven as a novel hypothesis beyond any reasonable doubt. This is because it relied on at least one major and uncertain assumption, *i.e.* that the rays of sunlight reaching Earth were indeed parallel. No-one knew, at the time, how could that assumption be confirmed, so more definitive proof was needed.

In 1492, the maritime explorer and navigator Christopher Columbus (1451–1506) proposed a western sea route to India, based on the assumption that the Earth was a sphere. Like many other seafarers of his time, he was frustrated with the long and dangerous route from Europe to India, which required sailing around the entire African continent in the times before the Suez Canal had eventually been constructed [4]. In Columbus’s time, navigational techniques, refined by contemporary astronomers, already used the positions of the Sun and stars in the sky and the understanding that the Earth was likely spherical. However, although Eratosthenes’s ancient estimates had been remarkably precise, a series of errors had since been made when converting old units of measurement into new ones. The series of mistakes led to incorrect assumptions regarding geographical latitudes, longitudes, and the sizes of the European and Asian continents. For instance, Columbus estimated that the distance from the Canary Islands on Europe’s western edge to Japan, *via* the ‘western route’ across the ‘great ocean’, was only about 3700 km [5], whereas the actual distance is 19 600 km. Columbus, therefore, was an incredibly lucky person. Had there not been an entirely unknown landmass in his pass, *i.e.* North and South America, his ships would have run out of food and drinking water during their journey to India. All aboard would likely have perished and drowned somewhere in the vast ocean stretching from Europe’s western shores to Japan’s eastern coast, encompassing both the Atlantic and the Pacific Ocean.

The discovery of a new continent was certainly an interesting new piece of information, but it still did not prove that the Earth was spherical. A new expedition was required, one organised by the Portuguese explorer Ferdinand Magellan (1480–1521), to obtain irrefutable evidence. Magellan’s expedition, undertaken from 1519 to 1522, was the first human journey from the Atlantic Ocean to the Pacific Ocean through what is now known as the Strait of Magellan, between Antarctica and South America. It was also the first known human crossing of the Pacific Ocean and the first circumnavigation of the entire Earth [6]. Magellan, however, was far less fortunate than Columbus; he did not live to see his great success celebrated, as he was killed during the Battle of Mactan in the Philippines [7]. Still, even that magnificent voyage, which his crew managed to complete without him, represented only indirect evidence of the Earth’s shape as a sphere. Earth could still have been an ovoid or even a cylindrical structure. For those who needed to see a sphere before they believed it, the first direct photographic evidence was gathered in 1946, at the onset of the ‘space race’ between the United States and the Soviet Union. That was when the first images from space arrived back to Earth’s surface, clearly showing the curvature of the Earth and its spherical shape [8].

Still, the proof that the Earth is round did not refute another deeply rooted belief based on our sensory perception: that the Earth is the centre of the Universe. Nicolaus Copernicus (1473–1543) was a Polish astronomer, mathematician, and Jesuit who worked during the Renaissance. He formulated the so-called heliocentric model of the Universe, in which he placed the Sun, rather than the Earth, at its centre. His most significant book, *De revolutionibus orbium coelestium* (‘On the Revolutions of the Celestial Spheres’), published just before his death in 1543, eventually became one of the cornerstones of the entire scientific revolution. His understanding of the universe was based on a set of seven hypotheses. Among them were the ideas that ‘the centre of the Earth is not the centre of the universe, but only the centre of gravity and of the Moon’s orbit’, and that ‘what appears to us as the motion of the Sun is not due to the Sun’s movement, but to the Earth’s movement, which orbits the Sun like any other planet’. He also concluded from the relationship between the Earth and the Sun that the Earth must have ‘more than one type of motion’ [9].

Yet even though these hypotheses were truly revolutionary, the question remained: how could they be tested? At the time, they could not be verified through experimentation. Copernicus had contemplated them for many years, but published his work only shortly before his death. As a result, the Church’s reaction was not as harsh as it might have been, primarily because Copernicus’s entire contribution remained purely theoretical. During that era, many other theories were also circulating and none of them had any experimental proof. However, in the early 17th century, the telescope was invented. Galileo Galilei (1564–1642), the Italian physicist, mathematician, engineer, astronomer, and philosopher, is often credited with this invention, although this is not entirely accurate. Nevertheless, Galileo developed the instrument independently of others and was the first to apply it to astronomy in 1609 [10]. Using this new measuring tool, the telescope, as an extended sense of vision, Galileo was able to confirm the hypotheses proposed by Copernicus. This, however, provoked a much harsher reaction from Church authorities, who banned all books supporting the Copernican system and tried Galileo, placing him under house arrest [11]. Copernicus and Galileo thus form a notable pair of great scientists: one formulated revolutionary new hypotheses that could not be tested at the time and the other developed and applied a measuring device to experimentally confirm those hypotheses.

Another pair of great scientists made enormous breakthroughs in a completely different field of science, though through a similar sequence of events. In their case, rather than trying to understand phenomena much larger than ourselves, like planets and the universe, they focused on phenomena far smaller than us. Infectious diseases caused mass death throughout human history, but few today can truly grasp how terrifying they must have been in an era when their cause was completely unknown. Epidemics would sweep through villages, claiming up to half of their population with each wave, and no one knew why they appeared, nor what they even were.

Girolamo Fracastoro (1476–1553) was an Italian physician and poet, educated in mathematics, geography, and astronomy, who proposed a compelling idea about the potential cause of infections. He was also a follower of atomist philosophy. In 1546, in his book *De Contagione et Contagiosis Morbis* (‘On Contagion and Contagious Diseases’), he hypothesised that epidemic diseases might be caused by particles – which he called ‘spores’ – that were simply too small to be seen and which could pass from one person to another. He suggested that these ‘spores’ could transmit infectious diseases over great distances through direct or indirect contact, or perhaps even without any contact at all [12]. While all those ideas may sound entirely reasonable today, at the time very few people imagined anything so small that it could not be seen with the naked eye. Fracastoro’s hypothesis could only be tested in the following century. In the Netherlands, another great scientist, Antonie van Leeuwenhoek (1632–1723), developed the microscope in 1670, which enabled yet another enhancement of the human sense of sight [13]. Using his microscope, he was astonished to discover tiny, living microorganisms in material taken from infected wounds, thereby confirming Fracastoro’s hypothesis.

If we linger a little longer on the perpetual battle against infectious diseases, we must also mention Edward Jenner (1749–1823), another exemplar of hypothesis-driven scientist. He was an English physician who introduced vaccination against smallpox, the world’s first vaccine. This innovation ultimately became responsible for saving more human lives than any other medical intervention in history [14]. Jenner began his work intrigued by an anomaly in existing knowledge that demanded explanation. He observed that girls who milked cows were immune to smallpox, whereas no one else in the village shared this immunity. His hypothesis was that the pus from cowpox, which the girls came into contact while milking, somehow provided protection against smallpox. Cowpox was a disease similar to smallpox in humans, although far less dangerous. Jenner conducted an experiment to test his hypothesis in 1796: he exposed an eight-year-old village boy to the cowpox material. The boy developed a fever and temporarily felt unwell, but did not contract a full-blown infection. Jenner then successfully tested his hypothesis on another 23 villagers and published his findings in 1798 in the book ‘An Inquiry into the Causes and Effects of the *Variolae Vaccinae*’. The medical and scientific communities took a long time to accept such unusual and unexpected findings, eventually doing so only in 1840, when the government introduced free cowpox vaccinations to protect the population from smallpox [15].

Two more scientists deserve mention for having developed elegant research designs to test their hypotheses. One conducted the first community-based epidemiological study, while the other carried out the first randomised clinical trial. The first was John Snow (1813–1858), an English physician and a pioneer in promoting the importance of hygiene for health. He investigated the source of a cholera epidemic in Soho, London, in 1854. Snow did not believe in the prevailing theory of ‘miasmas’, which blamed ‘bad air’ for all infectious diseases. He hypothesised that water, not air, might somehow be responsible. To gather evidence supporting his hypothesis, he interviewed many locals and carefully mapped all the cholera cases in London. Based on the distribution of those affected, he concluded that the public water pump on Broad Street (now Broadwick Street) was the source of the outbreak. Local authorities then shut down the pump, halting further spread of the disease. John Snow’s study is now considered foundational for the science of epidemiology [16].

James Lind (1716–1794) was a Scottish physician in the Royal Navy. He noted that scurvy caused more deaths in the British fleet than all their enemies combined. Scurvy is a disease caused by a deficiency of vitamin C, though the concept of vitamins was unknown in Lind’s time. Through careful observation of naval sailors, Lind developed the hypothesis that fruit, especially citrus, might cure scurvy. Long before Lind’s time, it had already been speculated that citrus fruits might help treat scurvy, but he was the first to systematically test this hypothesis. In 1747, after two months at sea, when scurvy had begun to appear on his ship, Lind divided the affected sailors into six groups. Each group was given the same basic diet, but with one different food supplement. The group that received citrus fruits recovered, while no improvements were seen in the other groups. Lind retired soon after the successful experiment, and in 1753, published his work, ‘A Treatise of the Scurvy’ [17].

Another researcher guided by hypothesis testing certainly deserves mention. His achievements stem partly from the scale and significance of his hypothesis, and partly from the tremendous effort he invested in collecting a large body of valuable evidence to support it. He was Charles Darwin (1809–1882), an English naturalist. His hypothesis was that all species and forms of life on Earth had evolved over a long period of time from common ancestors, through a process he called ‘natural selection’. His theory was so vast, encompassing all known space and time, that enormous personal effort was required to gather sufficient information in support of it. Although he suffered from various illnesses and even had severe seasickness, this did not prevent him from boarding HMS Beagle in 1831. The ship’s voyage around the world, aimed at improving maps of the South American coastline, lasted almost five years [18]. Darwin sought to spend as much of that time on land as possible, observing nature and gathering data to support his hypothesis. He studied geological features in different areas, collected plant and animal specimens, meticulously kept a journal of his observations, and regularly sent his results back to Cambridge. Only three decades after beginning his journey, in 1859, did he publish his theory of evolution, rich in various forms of supporting evidence, in the book ‘On the Origin of Species’ [19]. By the 1870s, the scientific community and much of the global population had accepted evolution as fact. The later emergence of the ‘Modern Synthesis’ (between 1930 and 1960) added many new findings that led to scientific consensus that natural selection was indeed the fundamental mechanism of evolution. In the 21st century, technological advances have enabled comparative genomic studies across species, providing even stronger evidence [20,21]. Darwin’s hypothesis, now supported by a vast array of data, has grown into a unifying theory of the life sciences that explains the diversity of life on Earth [22].

Another example of a researcher driven by a hypothesis who tested a hypothesis of a more limited scope, yet shared some similarities with Darwin, was the Norwegian ethnographer Thor Heyerdahl (1914–2002). Like Darwin, he was educated in biology, zoology, botany, and geography. He hypothesised that ancient peoples were able to undertake very long sea voyages, creating contact between geographically distant cultures, thus supporting the diffusionist model of cultural development. He believed that people from South America could have settled the Polynesian islands before Columbus’s voyages. To prove his hypothesis, he hand-built a raft and, after naming it ‘Kon-Tiki’ after the sun god of the ancient Incas, used it to sail 8000 km across the Pacific Ocean in 1947, from the western coast of South America to the Tuamotu archipelago in Polynesia. The raft was built from natural local materials using the same methods as the indigenous people, based on drawings left by Spanish conquistadors [23]. Interestingly, most anthropologists were not particularly convinced by the outcome of his experiment, but genetic research in the 21st century later showed that the inhabitants of Easter Island do indeed carry DNA markers characteristic of South American populations [24].

## WHEN HYPOTHESES ARE DIFFICULT TO TEST: THE CASES OF ALBERT EINSTEIN AND PETER HIGGS

It is possible to develop many highly elegant hypotheses that provide beautiful theoretical explanations for events and observations in nature. However, such hypotheses can be incredibly difficult to test in practice, simply because no experiment can be designed to do so, given the level of technological development at the time of their proposal. When Albert Einstein (1879–1955), a theoretical physicist originally from Germany, developed the general theory of relativity in the early 20th century and applied it to model the structure of the universe on a large scale, it was difficult to believe that the gravity of massive objects in space could actually bend the path of light from distant stars [25]. However, there was a way to test this experimentally, but it required waiting for a total solar eclipse. Scientists waited several years until, in 1919, Arthur Eddington (1882–1944) organised an expedition that confirmed that light rays do indeed bend in the vast expanses of space. Eddington recorded changes in the positions of stars near the Sun’s edge, visible only during a total solar eclipse, and showed that the Sun’s gravity deflects light from those stars. These observations were made simultaneously in the cities of Sobral in Brazil and São Tomé and Príncipe, an island nation off the west coast of Africa [26].

Einstein was quite fortunate because he had to wait only a few years for a suitable solar eclipse. In comparison, Peter Higgs (born 1929), a British theoretical physicist, had to wait for much longer. While trying to explain the origin of mass in elementary particles in 1960, he introduced the concept of spontaneous symmetry breaking – known as ‘the Higgs mechanism’ – into the Standard Model of particle physics. He predicted the existence of an entirely new particle, the Higgs boson, often referred to in popular science as ‘the God particle’ or ‘the most sought-after particle in modern physics’ [27]. At the time of his work, the technological capacity required to test his hypotheses gave Higgs virtually no hope that they would be confirmed within his lifetime. Therefore, it was nearly miraculous that, half a century later in 2012, a dedicated team of several thousand physicists working at CERN in Geneva announced that they had experimentally confirmed the existence of the Higgs boson [28].

The measuring device required to test Higgs’ hypothesis, the Large Hadron Collider, is the world’s largest and most powerful particle accelerator. It began its operations in 2008 and consists of a 27-km underground ring of superconducting magnets along with numerous additional accelerating structures. Two beams of high-energy particles are accelerated inside the ring to near-light speeds before being made to collide. The particle beams travel in opposite directions in separate vacuum tubes, guided by strong magnetic fields generated by electromagnets cooled to a temperature colder than outer space [29]. The Higgs mechanism is now widely accepted as a key component of the Standard Model of particle physics, without which certain particles would not have mass at all. Higgs had to wait more than 50 years to receive his Nobel Prize, which was shared with Belgian physicist François Englert in 2013 [30]. Still, he could consider himself far more fortunate than Copernicus or Fracastoro, who both died long before the invention of telescopes or microscopes that could confirm their revolutionary hypotheses about the world around us.

## WHEN HYPOTHESES CANNOT BE TESTED: THE CASE OF SIGMUND FREUD

In contrast to all the scientists mentioned so far, each of whom proposed specific novel hypotheses that required experimental testing, Sigmund Freud (1856–1939) developed an entire system of hypotheses to explain the psychological disorders of his patients. He was an Austrian neurologist who founded the field of psychoanalysis and proposed that the human psyche could be divided into three parts: the conscious, the unconscious, and the preconscious. He elaborated this model in the essay ‘Beyond the Pleasure Principle’ (1920) and further deepened it in ‘The Ego and the Id’ (1923). Furthermore, he proposed hypotheses suggesting that human behaviour is governed by two distinct, yet conflicting central drives: the life drive (libido or *Eros*) and the death drive (later called *Thanatos*); and that the libido is a form of mental energy encompassing processes, structures, and objects [31]. Experiments designed to test Freud’s hypotheses gave rise to a vast body of literature in the field, but most of the results remained inconclusive or were too difficult to interpret [32].

For this reason, Freud’s case can assist in understanding what is required to become a successful researcher within a hypothesis-driven science. He was undoubtedly an exceptional and charismatic intellectual who was able to develop numerous creative and novel hypotheses. He also created several new methods, such as the use of free association and transference, on which the new discipline of psychoanalysis was founded. Seemingly, he was following the path of the most successful scientists who developed both the revolutionary hypotheses and the methods to test them. Still, after all the scientific work in this area, most scientists will agree that his contributions remain questionable at best and that the discipline cannot be considered truly scientific in the full empirical sense.

Karl Popper (1902–1994), one of the greatest philosophers of science, believed that all ‘true’ scientific theories and hypotheses must be falsifiable. Upon analysing Freud’s work, Popper concluded that Freud had presented his psychoanalytic theories in forms that made them unfalsifiable. That is, no experiment could be designed that would produce data sufficient to reject Freud’s theories [33]. In his work ‘The Myth of the Framework’ [34], Popper wrote: 

‘*Any scientist who claims that their hypothesis is supported by experiment or observation should be prepared to ask themselves the following question: Can I describe any possible result of that experiment or observation which, if recorded, would refute my hypothesis? If not, then my hypothesis is clearly not an empirical theory… I also call this criterion the criterion of falsifiability or refutability. It does not imply that hypotheses which cannot be falsified are necessarily wrong. Nor does it mean that such hypotheses are unimportant. However, it does indicate that, as long as we cannot describe how a given hypothesis might be rejected, we must consider that theory to lie outside the realm of empirical science*.’

## WHEN HYPOTHESES ARE NO LONGER CRUCIAL FOR DISCOVERY: THE CASE OF GWAS CONSORTIA

The previous sections demonstrated that the historical development of science, discovery, and knowledge has largely progressed through the formulation of hypotheses, followed by careful experimentation and measurement to either confirm or refute the proposed hypotheses. However, the early 21st century saw an intriguing addition to this approach which feels different and challenges the traditional sequence of knowledge acquisition, hypothesis generation, data collection and experimental testing. The rise of large-scale datasets of unprecedented scale such as human biobanks and the growing storage and analytical capabilities of modern computers led to the emergence of new approaches to science. Instead of the knowledge and the hypothesis, the starting point for discovery can move to massive data repository themselves which were built without any specific or novel hypotheses. Very large number of analyses can then be conducted systematically in those datasets without a specific research question in mind. Hypotheses can then be generated *a posteriori*, *i.e.* after the systematic data analyses have revealed something previously unknown.

Among the most compelling examples of this new approach are genome-wide association studies (GWAS), which have transformed the way we explore the relationships between genetic variation and human health [35,36]. These GWAS arose from the convergence of several scientific breakthroughs: the sequencing of the human genome, the development of high-throughput genotyping technologies, the availability of large cohorts with genetic and phenotypic data, and the increase in digital memory and computational power needed to handle vast volumes of information. Unlike classical studies in genetics or medicine that would begin with a specific hypothesis based on previous knowledge such as ‘gene X might be involved in disease Y’, GWAS can be launched without any specific hypothesis on which genetic variants could be associated with various biomedical traits. Instead, millions of single nucleotide polymorphisms (SNPs) across the entire genome can be tested for their statistical association with a wide array of biomedical traits, such as height, cholesterol level, blood pressure, risk of diabetes, or presence of psychiatric conditions. The scale of this process is enormous: a single GWAS might involve testing millions of SNPs for association with hundreds of traits in a million human subjects. This creates a reversal of the traditional model: rather than starting with a hypothesis and seeking data to confirm it, GWAS analyses begin with massive amounts of data and allow statistically significant results to suggest hypotheses that can later be explored biologically [35,36].

This paradigm shift would have been inconceivable in the past. Yet the GWAS approach encountered an interesting new problem in testing the hypotheses: false-positive results, whereby the likelihood of finding associations that are false positive increases dramatically with the number of tests performed. This necessitates that statistical thresholds in GWAS studies be set at extremely conservative levels. The typical, historic thresholds for statistical significance required to reject the tested hypotheses needed to be replaced by remarkably low *P*-values, often below 10^−8^ or even smaller, to declare an association formally significant and account for the problem of multiple testing. Such levels of statistical confirmation can only be reached in incredibly large datasets. This prompted scientists from around the world who developed biobanks of realistic sample sizes – such as the ‘*10001 Dalmatians*’ which I began developing in Croatian island isolate populations in 1999 with Professors Harry Campbell and Alan F. Wright at the University of Edinburgh [37] – to join forces and merge all their data. Their collaboration generated large enough biobanks with hundreds of thousands, or eventually millions of subjects, and allowed sufficient ‘statistical power’ for credible findings to emerge. Such merging of many independently created datasets led to so-called ‘GWAS consortia’, which is the reason why most papers based on GWAS approach eventually had dozens, or even hundreds of co-authors. In addition to the requirement of a very large sample size when assembling biobanks of genetic and phenotypic data, other key requirements were the technological capacity to measure millions of genetic variants quickly and affordably, which was enabled by private companies that made remarkable advances in increasing the speed and accuracy of genotyping, while reducing costs dramatically [35,36].

When such associations emerge, they often point to regions of the genome that were not previously suspected to affect the biomedical trait of interest. This has led to many unexpected discoveries: for instance, associations between genetic variants and many disorders, syndromes, quantitative traits, diseases, and even social or cognitive characteristics. These findings have prompted researchers to work ‘backwards’: they would start with the statistically strongly confirmed signal of previously unsuspected association, trying to understand the underlying biology and how the function of an implicated gene might influence the trait in question. In a sense, the GWAS approach made it possible for researchers to arrive at correct answers before they had enough knowledge to ask the questions. This ‘reverse discovery’ represents a stark departure from traditional scientific reasoning, which moves forward from knowledge to prediction, and then to validation [35,36].

One of the most important findings of the GWAS approach was that traits most amenable to GWAS discovery tend to be polygenic, meaning that they are influenced by hundreds, or thousands, of small-effect variants spread across the genome. Our group in Edinburgh was likely the first to predict such finding based on empirical results from the ‘10001 Dalmatians’ dataset [38]. In polygenic model, no single variant determines whether a person will develop a complex disease; instead, it is the cumulative effect of many variants, each contributing a tiny increase in risk, that eventually contributes to disease development. This insight has eventually given rise to the concept of polygenic risk scores (PRS) which combine the effects of many SNPs into a single metric that estimates a person’s genetic predisposition to a trait or disease [39,40]. While PRS are not diagnostic, they represent a promising tool for personalised medicine, risk stratification, and early intervention, provided they are used responsibly and with full awareness of their limitations. Importantly, most GWAS studies have been performed in populations of European descent, raising questions about the transferability and equity of polygenic predictions across diverse populations; efforts are now under way to diversify the biobanks and ensure that discoveries benefit global health [40,41].

## WHEN HUMAN SCIENTISTS ARE NO LONGER CRUCIAL FOR DISCOVERY: THE CASE OF ALPHAFOLD AND DEMIS HASSABIS

In the grand trajectory of scientific progress, we have witnessed a transformation in how new knowledge is generated. From the solitary geniuses who were educated well enough to form creative novel hypotheses and methods to test them, to large teams of researchers using biobanks and computers to test millions of hypotheses simultaneously, the approach to generate scientific discovery has truly evolved. Yet perhaps the most dramatic transformation is the one that unfolded in recent years, in which artificial intelligence (AI), rather than human scientists, began to uncover truths about the natural world. One of the most prominent scientists standing in the centre of this transformation is Sir Demis Hassabis, the co-founder and CEO of DeepMind, whose team’s development of AlphaFold represents potentially the most ground-breaking shift in the history, philosophy, and practice of science.

In 2023, Sir Demis Hassabis and John Jumper, his collaborator at DeepMind, were awarded the Breakthrough Prize in Life Sciences for their work on solving the protein-folding problem, a scientific challenge that had eluded even the most advanced biological research for many decades [42]. They then shared the Nobel Prize in Chemistry in 2024 for creating an AI system capable of predicting the three-dimensional structure of proteins from their amino acid sequences with unprecedented accuracy. The award recognised a massive progress beyond the discovery itself, where science was not conducted through hypothesis-driven experiments nor data-driven associations, but rather by machines that were capable of deriving predictive patterns at a level that was far beyond what human cognition could possibly achieve [43].

To understand the significance of AlphaFold, the magnitude of the protein-folding problem needs to be appreciated. Proteins are complex chains of amino acids folded into three-dimensional shapes that determine their function. The sequence of amino acids is encoded in DNA and can now be read, but predicting how that chain would fold into a final spatial shape proved immensely difficult, as the number of ways in which this can occur is unimaginably large. Scientists attempted to use traditional scientific methods to solve this problem: they formed hypotheses about folding mechanisms, tested them with increasingly powerful experimental tools like x-ray crystallography, nuclear magnetic resonance, and cryo-electron microscopy. These techniques were time-consuming, costly, and required highly specialised expertise, so even the most advanced laboratories could only resolve the structure of a few proteins per year.

Sir Demis Hassabis, a neuroscientist and AI researcher fascinated by intelligence who had originally trained in cognitive science and neuroscience before completing a PhD in artificial intelligence at University College London, co-founded DeepMind in 2010. His vision was to create AI systems that could learn and reason like the human brain, having a ‘general artificial intelligence’. In 2016, DeepMind’s landmark success demonstrated that AlphaGo system can defeat the world’s best Go player using deep reinforcement learning. The focus then turned to protein folding problem, and in 2020, AlphaFold stunned the scientific community when it succeeded in predicting the structure of proteins [42,43].

This was a new kind of science: rather than being explicitly programmed with biological rules, AlphaFold was trained on large public databases of known protein structures using a form of deep learning. The system learned complex patterns of spatial relationships between amino acids, implicitly internalising principles of chemistry, physics, and evolutionary biology without ever being explicitly taught them. In effect, AlphaFold was not guided by any previously developed human theories, nor was it testing any hypotheses. It was set free to learn from massive data that were assembled with accuracy and then asked to predict the outcomes for all the other proteins. It succeeded in doing so with stunning precision.

This unique case represents a remarkable advancement of the scientific process. Human scientists would traditionally acquire knowledge, think of an interesting research question, develop a hypothesis, design an experiment, acquire data and test the hypothesis. In AlphaFold’s case, starting with an interesting research question (‘How do proteins fold?’) was common to what human scientists would do. Human scientists have also produced and curated the data, as well as trained the model and validated the results. However, the path to discovery was no longer transparent to them, because the understanding based on the data that lead to the key progress and all the novel discoveries were achieved by a machine. The implications of this milestone for the future of science are likely to be immense.

DeepMind released the predicted structures for nearly every known protein in the human genome by 2021 and then expanded its predictions to over 200 million proteins from more than one million species in 2022 [42–44]. Human scientists suddenly gained access to structural information that would have literally taken centuries to accumulate through traditional methods. This will stimulate and vastly accelerate scientific progress in many areas of biomedicine. With AlphaFold, science took a step beyond hypothesis-driven and data-driven approaches into what might perhaps be called ‘machine-inferred science’. In this approach, the machine is no longer a tool that improves human senses, creates new ones, or amplifies human reasoning. Instead, it becomes a reasoning entity itself, replacing humans in a crucial part of the scientific process and uncovering patterns that no human brain could. In this, it may not even be able to offer an interpretation or rationale that human scientists could understand, implying the limits of human cognition when it comes to understanding the processes that occur in nature.

This unforeseen progress is truly fascinating, but in addition to remarkable opportunities, it also brings serious challenges. The potential for acceleration of scientific discovery in many areas has become difficult to comprehend. The human inability to entirely follow and understand machine reasoning introduces new concerns, because it is difficult to review, evaluate, and trust any results that humans cannot explain. The deployment of AI will, therefore, not be without risks; it is not apparent how can systems with so much potential influence on science, medicine, and society be managed. AlphaFold might be understood as a proof-of-concept that AI may soon play a central role not just in participating in the key parts of the scientific process, but in taking over most, if not all the steps.

In the context of this editorial, AlphaFold represents a revolutionary new phase in the evolution of scientific inquiry. If traditional science was hypothesis-driven and GWAS consortia were data-driven, then AlphaFold marks the beginning of machine-driven discovery. It no longer requires previous knowledge or hypotheses to test, nor does it need human intuition to guide it. It may soon be able to do almost everything on its own, choosing its own path to discovery which may differ quite significantly from those humans traditionally used. Increasingly, it does not even require a human to be present in the moment when discovery occurs. Because of this, the perception of what will it mean to be a ‘successful scientist’ in the future will likely be undergoing substantial changes.

## WHEN A NEW FIELD OF SCIENCE IS BORN: THE CASE OF IDEOMETRICS

In my previous editorial that asked a question ‘what makes science successful?’ [45], I noted that new scientific fields can emerge when a novel tool or a method is developed that can be used for observations or measurements that generate novel type of data. Opportunity to study such new data can then lead to entirely novel hypotheses, while confirmation of such hypotheses can give rise to new fields of scientific discovery. In this section, I will present a personal experience of developing a novel scientific method which gained broad implementation: the Child Health and Nutrition Research Initiative (CHNRI) method for setting health research priorities. I will then explain how the insights from implementation of the CHNRI method eventually led to a novel hypothesis. Finally, I will explain how this hypothesis, combined with the new data, led to the development of a new field of science which I call ‘ideometrics’ – the scientific approach to generating, measuring, and prioritising ideas.

In a recent review, I tracked the evolution of the CHNRI method for setting health research priorities [46]. Funded by the World Bank and commissioned by the Global Forum for Health Research, it was first presented in 2006. I led an interdisciplinary group that worked on its development. Our aim was to tackle three key barriers to universal research priority setting: coping with an incredibly large number of competing research hypotheses, managing uncertainty about the outcome of their testing, and providing an opportunity for a fair, transparent, and acceptable consensus. We proposed solutions that addressed those challenges through a systematic process of generating ideas, careful definition of the context, fully transparent criteria that can discriminate between different research ideas, and crowdsourced scoring of many experts. Through this approach, the novel type of data that was generated were expert scores that measured a ‘collective optimism’ towards many ideas. Scores assigned to different criteria could be further weighted by non-experts, *e.g.* funders and stakeholders [46].

Early demonstrations of the usefulness of the CHNRI method soon led to its uptake by the World Health Organization, UNICEF, the World Bank, the European Commission, several other major agencies, and many governments that used it for national-level health research priority setting. Furthermore, many international scientific collaborations were formed to use the CHNRI method to set health research priorities for a specific challenge. Within a decade from its introduction, this method became the most widely used approach to health research priority setting [47]. The review of the first 50 conducted exercises identified its advantages: it was systematic, transparent, inclusive, flexible, simple, and it did not cost much to conduct [48].

The novelty of the crowdsourced data that the CHNRI method acquired, *i.e.* the sheets with expert scores for each research idea against each proposed criterion (which could be either ‘0’, ‘1’, ‘0.5’ or ‘blank’), enabled the first studies on quantitative properties of human collective knowledge and opinion. The experiments in human collective knowledge confirmed that the scores by experts will be much more accurate than those by non-experts [49]. Analyses of human collective opinion showed that there is a point of ‘stabilisation’ which occurs when 45–55 scorers are engaged, meaning they achieve very stable rankings that do not change with addition of further experts and their scores [50]. Further improvements of the CHNRI method introduced bootstrapped confidence intervals, an information-theory expert agreement metric, and clustering analysis to detect scorer sub-structures, strengthening its scientific rigor [51]. Recently, it integrated AI-based large language models (LLMs) as partners in all steps of the priority-setting process [51].

How did this novel method, which enabled generation of the new kind of data, lead to a novel hypothesis? Although the CHNRI method was initially used for setting health research priorities, it eventually became a near-universal tool for ‘measuring’ research ideas: it assigned them greater or smaller scores based on the crowdsourced expert input. From there, it did not take much to realise that the CHNRI method could eventually be used to ‘measure ideas’, no matter in which field and for what purpose [52]. As a result, years of CHNRI implementation led to a novel hypothesis: viewing the brain’s ‘perception of ideas’ as an underappreciated human sense [53]. Humans have developed their ‘major’ and ‘additional’ senses based on specific neuronal receptors, and information from those sensors is integrated and processed in the brain. However, the brain itself has not been considered a sensory organ associated with a particular sense. The CHNRI method revealed that the brain’s ‘perception of ideas’ has many elements of a sense where the brain is a sensory organ. The human brain is continuously being exposed to ideas which can be self-generated, triggered by information from other sensors, or introduced from the external world. Pursuing those ideas tends to drive most of human activity and requires prioritising between short-, mid-, and long-term investment of energy and time, similarly to the CHNRI method’s concept [53].

This hypothesis required defining ideas in the context of the brain’s sensory perception as ‘competing possibilities of purposeful activities that, if followed, would be expected to result in an alternative version of the future’ [53]. The brain’s sensory role is to continuously assess many competing ideas and prioritise between them based on certain inherent criteria, similarly as the CHNRI method does with research ideas. Those criteria, at the personal level, are motivational/emotional (‘attractiveness’), operational/rational (‘feasibility’) and outcome-related perspective (‘impact’). Interestingly, in a human, exposure of the brain to new ideas may instigate physiological and psychological responses, such as enthusiasm, excitement, or fear [53]. Large groups of people can be mobilised by shared ideas, and their brains can respond to them with excessive enthusiasm that can become fanatical. Based on that notion, it becomes apparent that prioritisation of ideas at the personal level is affected by cultural and educational history, experience, and cognitive abilities. Therefore, brain’s ‘sense of ideas’ may be sharpened and made more successful in recognising ideas that would lead to better future outcomes through an increased level of expert knowledge and experience. Consequently, disinformation is remarkably dangerous because it negatively affects the brain’s ‘perception of ideas’, and ability to prioritise the most promising ones.

From the hypothesis of the brain’s role as a ‘sensor of ideas’, it emerged that its stored information about the nature and rules of the external world – which is analogous to the very precise definition of the context in the CHNRI method – becomes central to optimal functioning of this sense. In a subsequent paper, I explained that not all information that the brain collects and stores that can be used to the purpose of prioritising ideas is of equal value [54]. ‘Valuable information’ shifts probabilities assigned to our hypotheses in the most meaningful ways and significantly alters our knowledge and understanding of our context. ‘Valuable information’ will assist the brain in prioritising the ideas most likely to lead to favourable outcomes and improve decision-making. Interestingly, any available information can become ‘valuable’ only in the presence of a conscious observer. Similarly, disinformation needs conscious observers to distort the shared understanding of the context, mislead activities, and prompt observers to prioritise irrational ideas, exploiting the greater intensity of the brain’s response to unexpected surprises over the expected truth [54]. I proposed that ‘valuable information’ has some inherent characteristics: ‘relevance’ (being related to observers’ beliefs); ‘credibility’ (being trusted by its observers); and ‘leverage’ (having a decisive influence on the observers’ future actions). I then hypothesised that the future success in many human activities may not necessarily be linked to gathering the largest amount information, but rather identifying, generating, and acting upon the information that is ‘most valuable’ [54].

The proposed hypotheses of the brain’s ‘sense of ideas’’ and the ‘value of information’ which the brain uses to prioritise ideas could lead to further qualitative and quantitative experiments, establishing what I called ideometrics – the science of generating, evaluating and prioritising ideas. In the next step, in collaboration with Sir Aziz Sheikh, I tried to identify methodological approaches that have been used in many areas of human activity to assist the generation, evaluation, and prioritisation of ideas. We identified over 70 different methodological approaches and positioned and categorised them within the larger integrative framework of ideometrics [55]. We also showed that ideometrics is a falsifiable scientific field that offers predictive power and a cumulative research agenda, which makes it analogous to econometrics, bibliometrics, psychometrics, or decision sciences. It can test hypotheses about future performance of competing ideas, while its methods are increasingly evolving into rigorous, standardised, and quantitative tools supported by statistical inference and AI. It is possible to assess the scientific footprint of each of the >70 methodological approaches, develop formal reporting guidelines for future ideometrics studies, and conduct empirical comparisons between methods used to address the same challenges [55].

Ideometrics could have many practical applications: it can address the scarcity of time, energy, capacity, and resources in the presence of many competing ideas and identify those most likely to achieve their stated aims based on an objective and scientific approach, rather than intuition [55]. Its application in governmental activities and companies with a focus on scarcity could eventually position it within the field of economics, while its application to address the needs of entire societies could position it as an independent and highly interdisciplinary science. Ideometrics differs from adjacent fields of science, such as decision sciences, innovation studies, behavioural economics, philosophy of science, or management studies, because its scope is the entire lifecycle of ideas. Furthermore, it operates at a meta-level across contexts and independent of domains and offers comparative studies and validation of idea-processing methods, regardless of their discipline of origin.

The brain’s ‘sense of ideas’ might also contribute to the fields of evolutionary biology and studies of consciousness. Namely, most biological processes in living beings occur automatically, based on instructions encoded in the genetic material: from cell divisions to the function of the liver, kidneys, endocrine glands, digestive tract, or bones. There does not seem to be conscious control over the vast majority of the processes within any living being. Conscious beings, such as humans, can only control their voluntary musculature. Therefore, if we consider consciousness a complex trait that integrates several cognitive functions, an important evolutionary advantage of developing consciousness would be the possibility to exercise the brain’s ‘sense of ideas’ in order to dramatically reduce ‘informational entropy’ of all possible future states of a conscious being, steering it towards a narrow range of outcomes that are perceived as more favourable for its survival. This is achieved in a unique way: by simulating future outcomes that could result from following different ideas and then using voluntary musculature to make those outcomes a reality, while collapsing many alternative possibilities.

As a result, so-called ‘visionaries’ may achieve remarkably favourable personal futures that are entirely unlikely – or even implausible – such as building and owning incredibly successful multinational companies. To achieve this, they need to be exceptionally knowledgeable and well-informed about the context, so that they could follow a long sequence of really good ideas. Similarly, our entire species followed a sequence of remarkable ideas to land astronauts on the Moon and bring them back alive – an outcome entirely implausible to ever happen by chance, which is why it remained out of reach for all other species that ever inhabited the Earth.

Therefore, most humans use their ‘sense of ideas’ to gravitate towards simulated futures where threats to their survival should be minimised and chances of reproduction, prolonged existence, and well-being should be maximised. They try to earn more money, buy homes in safer neighbourhoods, drive bigger and safer cars, and avoid health risks. They also try to ensure that their children are educated at good schools, so that they could also understand the context quite well, leading them to exercise their ‘sense of ideas’ successfully towards a better future. Interestingly, one important prerequisite for the ‘sense of ideas’ to be exercised is the existence of ‘time’ and ‘space’, so that the ‘future’ can be planned and changed by voluntary movements in time and space, following the prioritised ideas. Therefore, an intriguing question is whether ‘time’ and ‘space’ are a ‘built-in’, endless axes of an internal ‘coordinate system’ within the conscious mind which are needed to plan and execute the prioritised ideas in the external environment. If this is the case, then it will need to be clarified whether ‘time’ and ‘space’ also exist outside of the mind in the same shape and form as we ‘feel’ them subjectively.

The ‘sense of time’ is needed for an internal ordering of events by a conscious mind that exercises its ‘sense of ideas’ based on its understanding of the cause-and-effect relationships in the external environment. When a conscious being can encode memory, analyse observed events, anticipate outcomes, perceive changes, and then act to avoid random and potentially dangerous or costly future outcomes, relying on the sense of ideas to identify preferred paths to future, ‘time’ and ‘space’ become the subjective categories that can accelerate and decelerate, expand and contract, often based on the person’s age, emotional state, or even in dreams. Therefore, our perception of time and space seems intertwined with our consciousness, dreams, and our ‘sense of ideas’. Understanding their relationship may become an interesting line of further research in neuroscience.

Based on this editorial’s content up to this point, a question could be posed about what will it take for ‘ideometrics’ to succeed as a new field of science? Its novel theoretical foundations have already been introduced [53–55]. In the next stage, the mathematical and statistical tools that could specifically support this field will need to be developed. Using the CHNRI method as the most advanced tool within this emerging field, optimal methodological solutions will be required for all three stages of the ‘idea cycle’:

– to generate ideas by addressing challenges of relying on human *vs*. AI-based ideation, expressing ideas mathematically based on the text they contain, studying the level of their similarity based on text processing, finding the ‘saturation points’ where novelty of further proposed ideas is reduced, solving the challenge of reducing the number of proposed ideas based on text processing while retaining the maximum amount of information, studying quantitative properties of human and LLM-based collective ideation, and defining saturation points;

– to evaluate ideas by addressing the problem of finding the most useful criteria and weighting them appropriately, optimising the scoring process and the number of scorers, improving related statistical analyses to determine confidence intervals and stability of scores, optimising agreement statistics between the scorers to identify the most controversial ideas, and checking the internal structure and interconnectedness of scorers to avoid biased scores;

– to prioritise ideas by solving the problem of optimised investment portfolio to support the optimal mix of ideas, and development the novel approaches to comparing human-based and AI-based prioritisation.

Then, the CHNRI method will need to be modified to allow its repurposing for the use by governments and companies, and for societal needs. Research grants will be required to support both the theoretical progress in addressing the above questions and for the expanded implementation of ideometrics in different context to achieve widespread use and acceptance. Finally, novel methods will also be required to evaluate implementation of ideometrics and demonstrate that it leads to more favourable outcomes than the alternative approaches to prioritising ideas.

## WHAT MAKES A SCIENTIST SUCCESSFUL?

What conclusions can we draw from the examples of successful scientists provided in this editorial? Clearly, science progresses from the unknown toward the known by building on existing knowledge, formulating new hypotheses, and conducting experiments involving measurement. Instruments based on technologies available to scientists in their historic context generated new information, which then enabled the confirmation or refutation of their hypotheses. In the more distant past, the scientific profession was available to only a small proportion of individuals within a community – those who had access to universities and libraries. Their success depended on their own abilities and motivation to develop and test new hypotheses.

Today, science is advancing faster than ever before. This is partly because the first step, which is the access to the entirety of human knowledge, has become available to almost anyone via the internet and its resources, such as Wikipedia [56] or AI-based LLMs. Then, massive publicly available repositories of ‘big data’ exist on the internet to enable ‘open science’ and allow for the simultaneous testing of many hypotheses without the need for prior knowledge, much like with GWAS. For those engaged in the field of AI, some entirely new ways of doing science are also becoming available.

So, what makes an individual scientist ‘successful’ in such a rapidly changing context? Historically, one path to recognition was through the development of a new measurement tool that provided humans with a novel ‘sense’ for observing and studying nature or enhanced an existing sense, thereby opening the door to new dimensions of inquiry and enabling hypothesis testing. Many great scientists are remembered today for having developed a new measurement device or introducing a new method, after which their fields of science were born or existing ones rapidly evolved. Those scientists were recognised even though they may not have proposed any revolutionary new hypotheses themselves. Others are remembered for their highly creative and innovative use of existing knowledge for developing new hypotheses that, once confirmed, led to an entirely new way of thinking and major progress, explained prior observations that did not fit the prevailing hypotheses, or fundamentally changed the perspectives on their scientific domain. Those scientists were not necessarily involved in the design and implementation of the experiments that confirmed their hypothesis but are credited with the breakthrough idea.

Finally, perhaps the greatest among the giants of the history of science were those who not only developed the revolutionary hypotheses but also created the measuring devices and then designed and conducted the experiments that generated the information needed to confirm their hypotheses. Yet from the later sections of this editorial, it also becomes evident that those traditional approaches to achieving success in science may soon become outdated. The era of computers, ‘big data’, and AI may entirely change the processes through which the new knowledge is generated and the rules of what counts as individual’s contribution. It is difficult to predict whether human scientists will remain as important in those processes. Those who manage to adapt most quickly and skilfully to this new and rapidly evolving environment may then be credited as the truly successful scientists of the 21st century.
